# Morphological Engineering of Electrospun Recycled Polyethylene Terephthalate (r-PET) Nanofibrous Membranes for Sustainable Air Filtration: A Critical Review

**DOI:** 10.3390/membranes16090286

**Published:** 2026-08-28

**Authors:** Wei Lin Ng, Abu Bakar Sulong, Eng-Poh Ng, Soon Huat Tan

**Affiliations:** 1 School of Chemical Engineering, Engineering Campus, Universiti Sains Malaysia, Nibong Tebal 14300, Pulau Pinang, Malaysia; 2 Faculty of Engineering and Built Environment, Department of Mechanical and Manufacturing Engineering, Universiti Kebangsaan Malaysia, Bangi 43600, Selangor, Malaysia; 3 School of Chemical Sciences, Universiti Sains Malaysia, Gelugor 11800, Pulau Pinang, Malaysia

**Keywords:** recycled polyethylene terephthalate (r-PET), electrospinning, air filtration, nanofibrous membranes, morphology engineering

## Abstract

The growing demand for high-performance air filtration materials, coupled with increasing concerns over plastic waste accumulation, has accelerated interest in sustainable filtration technologies. Recycled polyethylene terephthalate (r-PET) has emerged as a promising feedstock for electrospun nanofibrous membranes due to the abundance, low cost, and sustainability of this plastic waste feedstock. The filtration performance of r-PET membranes has been demonstrated to be comparable to that of conventional virgin polymer filters. This review critically examines recent developments in electrospun r-PET nanofibrous membranes for air filtration applications, with particular emphasis on the role of membrane morphology in governing filtration performance. Unlike previous reviews that primarily summarize electrospinning techniques or recycled polymer applications, this review critically evaluates how membrane morphology—including fiber diameter, pore architecture, bead-on-string structures, and multilayer configurations—governs filtration performance in electrospun r-PET membranes. Evidence suggests that rational morphological engineering plays a more decisive role than polymer chemistry in overcoming the conventional filtration efficiency–pressure drop trade-off. Finally, future research opportunities in scalable manufacturing, environmentally benign processing, and artificial intelligence-assisted membrane design are discussed to support the development of next-generation sustainable air filtration media.

## 1. Introduction

Air pollution is one of the biggest environmental health crises of the twenty-first century. According to the World Health Organization, ambient air pollution contributes to approximately 4.2 million premature deaths annually, and fine particulate matter (PM_2.5_, aerodynamic diameter ≤ 2.5 μm) has been identified as the major cause of these deaths [1]. The morbidity and mortality of cardiovascular disease, respiratory diseases, and neurotoxicity have been closely linked to exposure to PM_2.5_ by epidemiological evidence [2]. PM_2.5_ can pass through the lung gas–blood barrier and directly enter brain tissue through the olfactory nerve, resulting in generalized oxidative stress and inflammation leading to cerebrovascular damage and neurological impairment [3]. The massive rise in industrialization, fossil fuel combustion, the increasing volume of vehicle emissions, and the intensification of agricultural activity have all contributed to increasing PM_2.5_ concentrations dramatically above the WHO recommendation of 5 μg/m^3^ in many parts of Asia, Africa, and the Middle East [4]. Concurrently, indoor air quality has become a critical issue. Populations spend approximately 90% of their time indoors [5], where levels of pollutants frequently exceed those found outdoors due to sources such as infiltrating outdoor air pollution, building materials, personal care products, and cleaning agents. The United States and Canada had an annual average PM_2.5_ level in the year 2023 that was 39% higher than the safe air level recommended by WHO (5 μg/m^3^) and people spend more than 1200 h per year breathing air above safe limits [6]. This represents the double burden of outdoor and indoor air pollution and has created urgent demand for scalable, high-performance air filtration technologies suitable for household, healthcare, and industrial settings.

Mechanical fibrous filtration remains the dominant commercial approach for particulate removal, serving as a central component of heating, ventilation and air conditioning (HVAC) infrastructure, cleanroom environments, and personal respiratory protection. However, conventional microfiber-based media, such as glass fiber and melt-blown polypropylene filters, are restricted by a fundamental efficiency–pressure drop trade-off that limits their performance ceiling [7]. Electrospun nanofibrous membranes have proven to be an attractive structural solution; they present a higher specific surface area, higher inter-fiber porosity, and enhanced slip-flow characteristics that collectively improve particle capture while reducing airflow resistance [8]. Electrospinning has become the preferred fabrication route for membranes owing to its operational simplicity, material versatility, and scalability. Electrospun membranes fabricated from polymers including polyvinylidene fluoride (PVDF) [9], polyacrylonitrile (PAN) [10], polyamide (PA) [11], and polyimide (PI) [12] have each demonstrated strong filtration performance. However, the environmental impact, raw material cost, and long-term durability vary among these electrospun polymer systems [13]. Among the polymers used for air filters, polyethylene terephthalate (PET) occupies a distinctive position. PET combines excellent mechanical strength, broad chemical resistance, dimensional stability across elevated temperatures, and proven durability under high cyclic airflow, contributing to a long service life in challenging filtration conditions [14]. In particular, PET is the world’s most widely produced polyester, with global output of more than 90 million tonnes per year, mostly used in single-use beverage packaging and textile applications [15]. Over 90% of the approximately one million PET bottles sold every minute end up in landfills or oceans [16], indicating that there is a critical need to implement new management strategies that redirect this waste stream toward high-value applications. Although the PET bottle collection in North American PET achieved 39.2% in 2024, the global recycling rate is still insufficient to address the scale of PET waste generated [17]. The conversion of waste PET into high-value filtration media therefore offers a dual opportunity: reducing plastic waste accumulation while meeting growing demand for sustainable filtration materials. This is a direct embodiment of circular economy principles. Research into electrospinning of recycled PET (r-PET) from beverage bottles and textile waste has shown that it is viable to produce nanofibrous membranes with filtration performance comparable to or exceeding that of virgin polymer membranes [18,19]. Optimization of concentration, flow rate, and voltage in r-PET electrospinning has yielded uniform fibers with average diameters of approximately 329 nm and collection efficiencies of 95.65–99.99% [20], while ultrafine fibers (41 nm) produced from recycled textile PET fiber waste achieved efficiencies approaching 100% following electret charging, a post-treatment that embeds a permanent electrostatic charge to enhance particle capture [21]. r-PET has been proven to be a viable material for nanofibrous filtration, but most investigations have revealed that filtration performance is critically governed by membrane morphology rather than polymer choice alone [8,22,23]. Fiber diameter, porosity, pore size distribution, and higher-order structural features, including bead-on-string architectures and bilayer configurations, substantially modulate both particle capture and airflow resistance [16,24]. Previously, bead-on-string structures were regarded as undesirable defects arising from unstable jet formation during electrospinning. However, recent studies have shown that these structures, when deliberately engineered, can simultaneously achieve higher filtration efficiency and lower pressure drop compared with conventional uniform nanofibrous membranes [16]. Bilayer systems integrating fine-fiber and coarse-fiber layers combine two filtration mechanisms: mechanical interception and electrostatic attraction to achieve high filtration efficiency with low pressure drop, with demonstrated quality factors up to 0.064 Pa^−1^ [24].

Despite these developments in r-PET electrospinning and fiber morphology engineering for air filtration, few recent reviews have integrated r-PET electrospinning with morphology engineering for air filtration. Existing reviews addressing electrospun PET filtration have either focused on virgin polymer systems [25], briefly mentioned recycled feedstocks as one application among many without dedicated in-depth treatment [26], or examined morphology engineering strategies broadly across polymer systems without specifically addressing recycled PET or the sustainability dimension [27]. The gap between r-PET electrospinning and morphology-driven filtration design is particularly significant given the accelerating convergence of circular economy imperatives and advanced materials engineering in filtration research. The present review addresses this gap by providing a critical and integrated analysis of electrospun r-PET nanofibrous membranes for air filtration. The fundamental mechanisms of fibrous filtration and electrospinning process physics are discussed in brief, followed by a critical assessment of PET and r-PET as filtration feedstocks. The central contribution lies in the systematic examination of morphology-engineered PET filtration systems, specifically bead-on-string architectures, spider-net structures, and bilayer designs as structural approaches for overcoming the efficiency–pressure drop trade-off. Finally, persistent challenges and prospective research directions are identified to guide the development of next-generation sustainable filtration media.

## 2. Fundamentals of Air Filtration

**Figure 1:**
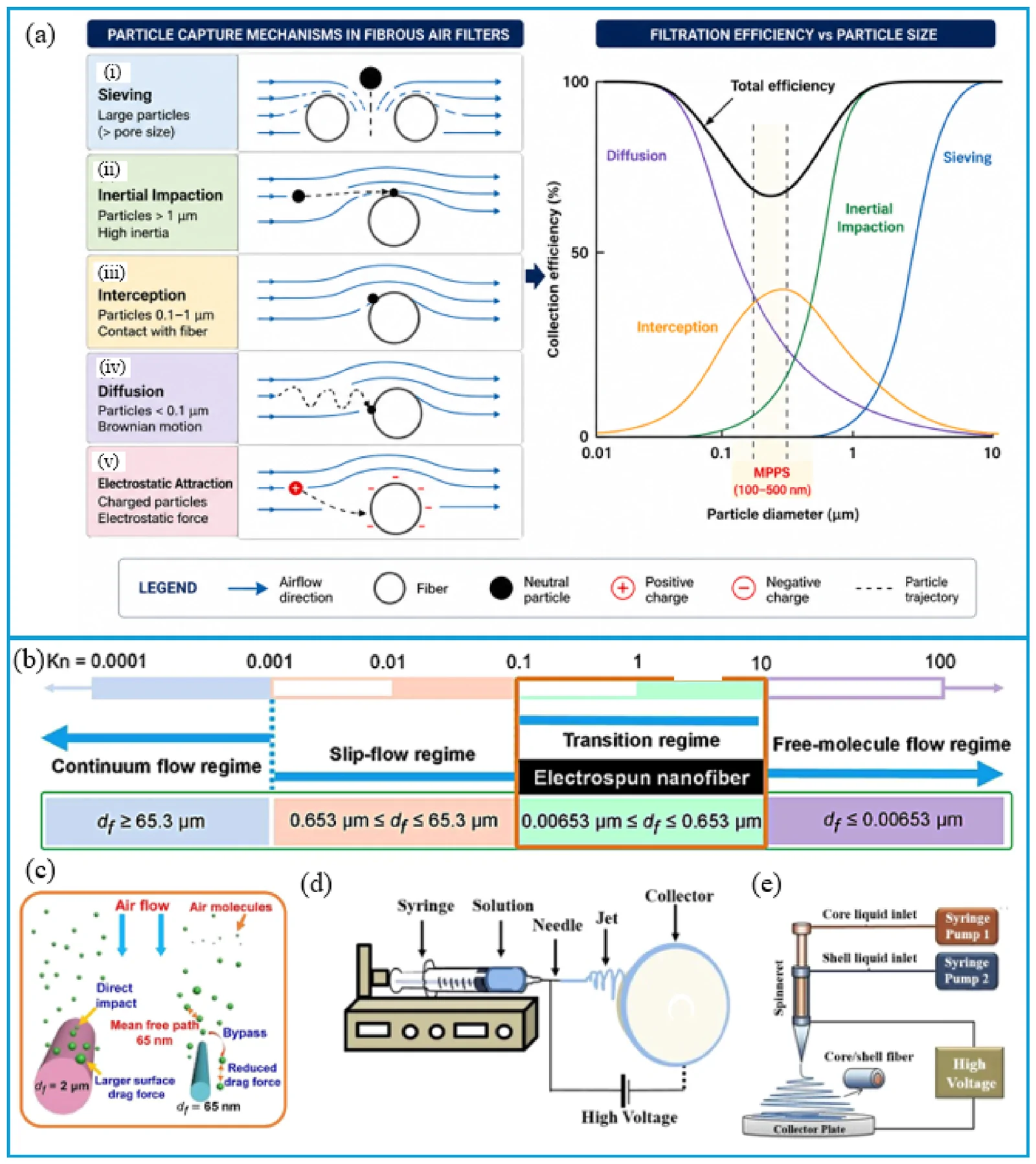
(**a**) Principal particle capture mechanisms in fibrous air filters include sieving, inertial impaction, interception, diffusion, and electrostatic attraction, together with the corresponding filtration efficiency–particle size relationship highlighting the most penetrating particle size (MPPS). (**b**,**c**) Illustration of the slip-flow effect in nanofibrous filters, showing the transition from the continuum flow regime to the slip-flow regime as fiber diameter decreases, resulting in reduced aerodynamic drag and lower pressure drop [31]. Schematic representation of (**d**) conventional electrospinning and (**e**) coaxial electrospinning setups used for the fabrication of nanofibrous filtration membranes [7].

Mechanical fibrous filters remove airborne particles through five primary mechanisms: sieving, inertial impaction, interception, diffusion, and electrostatic attraction, as illustrated in Figure 1a. The contribution of each mechanism depends on particle size, airflow velocity, and filter structure. Large particles are mainly captured through sieving and inertial impaction, whereas ultrafine particles are captured through diffusion. The combined action of these mechanisms produces the lowest filtration efficiency at a specific particle size known as the most penetrating particle size (MPPS) [28]. For many fibrous filters, the MPPS is typically reported between 0.1 and 0.5 μm [29]. Particles within this size range are the most difficult to capture and therefore represent a critical benchmark for evaluating filtration performance. The performance of a filter is commonly assessed using filtration efficiency (E) and pressure drop (ΔP). Filtration efficiency describes the fraction of particles removed from the incoming air stream, whereas pressure drop reflects the resistance encountered by airflow passing through the filter. A low pressure drop is desirable because it reduces energy consumption during operation. To evaluate both parameters simultaneously, the quality factor (QF), as shown in Equation (1), is frequently used. The QF combines filtration efficiency and pressure drop into a single performance metric. A higher QF indicates a more efficient filter with lower airflow resistance. A major challenge in air filtration is the inherent trade-off between filtration efficiency and pressure drop. Increasing particle capture often requires denser filter structures, which restrict airflow and increase energy demand. Consequently, considerable research has focused on electrospun nanofibrous membranes, which offer high surface-area-to-volume ratios and tuneable porous structures capable of improving filtration performance while maintaining acceptable airflow resistance [7].

(1) $$QF=-\frac{\ln\left(1-E\right)}{\Delta P}$$

Electrospun nanofibers address this trade-off through the slip flow effect [30,31]. As illustrated in Figure 1b,c, when the fiber diameter approaches the mean free path of air molecules (~66 nm), the Knudsen number (Kn = λ/df) enters the slip-flow regime. Under these conditions, airflow at the fiber surface no longer obeys the classical no-slip boundary condition, resulting in reduced viscous drag and lower pressure drop. Previous computational and experimental studies have confirmed that decreasing fiber diameter can enhance filtration efficiency while simultaneously benefiting from slip-flow-induced reductions in airflow resistance [32,33].

## 3. Fundamentals of Electrospinning

Electrospinning processes produce nanofibers through four key stages. The formation of a Taylor cone at the needle tip, ejection of a charged jet in a straight line, stretching of the jet into finer diameters under whipping instabilities, and finally fiber solidification and deposition on the collector. The process collectively yields continuous fibers with diameters from tens of nanometres to several micrometers [34]. Figure 1d depicts conventional single-needle electrospinning, in which a single polymer solution is drawn from one nozzle to form solid, uniform fibers. In contrast, Figure 1e shows coaxial electrospinning, where two concentrically arranged nozzles simultaneously deliver a core solution and a shell solution, producing core–shell fibers. This configuration enables the encapsulation of functional or sacrificial core materials within a protective shell, allowing additional control over fiber composition, degradation behavior, and functionality beyond what conventional electrospinning can achieve [35]. The simplicity, material versatility, and scalability to roll-to-roll formats have established electrospinning as the dominant nanofibrous filter fabrication technique [8]. Fiber morphology is governed by solution parameters (concentration, viscosity, conductivity), operational parameters (voltage, tip-to-collector distance, flow rate, deposition time), and ambient conditions (humidity, temperature). Among these, the polymer concentration is the most critical, as it determines solution spinnability through its effect on viscosity and surface tension. At low polymer concentrations, surface tension dominates over viscoelastic forces and produces beaded or bead-on-string structures, while optimal concentrations yield uniform bead-free fibers [34].

## 4. Polyethylene Terephthalate as a Filtration Material

The selection of suitable polymeric materials critically influences the performance, durability, cost, and sustainability of electrospun air filtration membranes. Among a wide range of polymers, polyacrylonitrile (PAN) and polyvinylidene fluoride (PVDF) remain the most extensively studied owing to their excellent electro-spinnability and high filtration efficiencies [36]. Electrospun PAN membranes have demonstrated filtration efficiencies of 99.27% with pressure drops as low as 33 Pa, attributed to their small pore size, high porosity, and specific surface area of 42 m^2^/g [36]. Meanwhile, PVDF has been extensively developed for its piezoelectric-enhanced electrostatic capture. A double-layer multiscale PVDF/FPU nanofiber membrane with ultrafine fiber diameters (~30 nm) has achieved 99.98% interception efficiency for PM_0.3_ at 67 Pa, with filtration performance maintained above 98.73% after 10 wash cycles [37]. However, increasing concerns regarding the environmental persistence of fluorinated polymers and their relatively high raw material cost constrain large-scale PVDF implementation [38]. Biopolymer-based materials such as cellulose and chitosan offer attractive sustainability advantages, yet their practical application remains constrained. Cellulose-based materials contain large numbers of hydrophilic groups that make them susceptible to humidity, and this moisture sensitivity promotes bacterial and fungal growth that greatly affects filter structure and performance under real operating conditions [39]. Chitosan similarly presents electrospinning difficulties due to its high viscosity and the formation of strong hydrogen bonds in a three-dimensional network that hinders polymeric chain mobility under an electric field, requiring blending with a spinnable polymer such as PVA to achieve adequate fiber formation [40]. These trade-offs have sustained interest in synthetic polymers that combine high performance with practical durability.

Polyethylene terephthalate (PET) has emerged as one of the most promising candidates for sustainable air filtration [20]. PET is a semi-crystalline thermoplastic polyester widely used in beverage packaging, textile fibers, engineering plastics, and consumer products. PET possesses high mechanical strength, thermal stability, chemical resistance, and dimensional stability that enable PET-based membranes to withstand prolonged operation under varying environmental conditions [20]. As electrospun PET fiber diameter decreases from 3.25 µm to 1.27 µm, mechanical strength increases from 3.2 to 4.0 MPa while collection efficiency reaches up to 100% at a low pressure drop of 212 Pa, demonstrating that structural refinement simultaneously improves both mechanical and filtration performance [18]. Furthermore, electrospun PET fibers exhibit significantly higher mechanical strength and ductility compared to many alternative filter polymers, owing to PET’s semicrystalline nature and superior polymer chain interactions, which maintain structural stability under cyclic airflow conditions [41]. Compared with other electrospinning polymers, PET offers a unique balance between performance and practicality, combining mechanical robustness, economic viability, and environmental sustainability. Table 1 summarizes the advantages and limitations of common electrospinning polymers employed in air filtration research, highlighting the distinctive position of PET and r-PET. While conventional polymers such as PAN, PVDF, PU, PA and cellulose-based materials each offer trade-offs between filtration performance and sustainability, r-PET uniquely combines the mechanical and chemical robustness of virgin PET with the added benefits of waste valorization and circular economy compatibility, making it a particularly promising candidate for sustainable air filtration applications. Consequently, increasing research efforts have shifted from merely investigating PET as a filtration material toward exploiting the structural tunability and recyclability of its post-consumer form, prompting direct comparison with other recycled thermoplastics considered for the same application.

Recycled polypropylene (r-PP) and recycled polyvinyl chloride (r-PVC) are the two recycled polymers most frequently considered alongside r-PET for electrospun air filtration. Both polymers have demonstrated genuine promise: r-PP nonwovens are well established in filtration via melt electrospinning and melt-blowing routes [42], and electrospun r-PVC membranes have achieved filtration efficiencies above 90% for PM_0.4_ and PM_2.5_ [43]. Within this context, r-PET possesses several favorable characteristics, including its solubility in suitable solvents, molecular structure, and processability, which make it particularly suitable for solution-based electrospinning. It dissolves readily in established solvent systems such as HFIP and TFA/DCM at room temperature, whereas PP’s limited solubility generally favors melt-based processing over solution electrospinning [42]. r-PET also retains much of the mechanical performance of virgin PET [18,44], and benefits from comparatively mature bottle- and textile-collection infrastructure [17]. PVC, meanwhile, is a chlorine-containing polymer, the recycling of which requires additional consideration of plasticizer and stabilizer additives, as well as chlorine-related by-products generated during thermal processing [45]. Table 1 summarizes the advantages and limitations of common electrospinning polymers used in air filtration research, with particular emphasis on the distinctive characteristics of PET and r-PET.

**Table 1:** Comparative overview of common electrospun polymers for air filtration.

Polymer *	Advantages	Limitations	Sustainability	Applications	Ref.
PAN	Excellent electro spinnability, high filtration efficiency	Petroleum-derived, limited recyclability	Moderate	HEPA filters, PM removal	[36,46,47]
PVDF	Strong electrostatic/piezoelectric properties, high chemical resistance	Fluorinated polymer environmental persistence, high cost	Moderate	Electret filters, high-efficiency filtration	[37,48]
PU	Flexible, stretchable, low pressure drop	Lower thermal stability	Moderate	Wearable filters, face masks	[49]
PA (Nylon)	Good mechanical strength and durability	Moisture sensitivity	Moderate	Industrial filtration	[47]
Cellulose	Renewable, biodegradable	Poor moisture resistance, low strength, susceptibility to bacteria/fungi growth	High	Sustainable filtration	[50,51]
Chitosan	Biodegradable, antimicrobial activity	Requires blending with PVA or CA for adequate electro spinnability and mechanical strength	High	Antimicrobial filtration	[52]
PET	Excellent mechanical strength, thermal stability, chemical resistance, low cost, established recycling infrastructure	Requires aggressive solvent systems (HFIP, TFA/DCM)	High	HVAC and industrial air filtration	[53,54,55]
r-PET	Waste valorization, low material cost, circular economy compatibility, filtration performance comparable to virgin PET	Feedstock quality variability	Very high	Sustainable air filtration and HVAC applications	[19]

* Abbreviations: polyurethane (PU); polyamide (PA); polyvinyl alcohol (PVA); cellulose acetate (CA); hexafluoroisopropanol (HFIP); trifluoroacetic acid (TFA); dichloromethane (DCM); high-efficiency particulate air (HEPA).

## 5. Recycled Polyethylene Terephthalate (r-PET): Transforming Plastic Waste into High-Performance Filters

**Figure 2:**
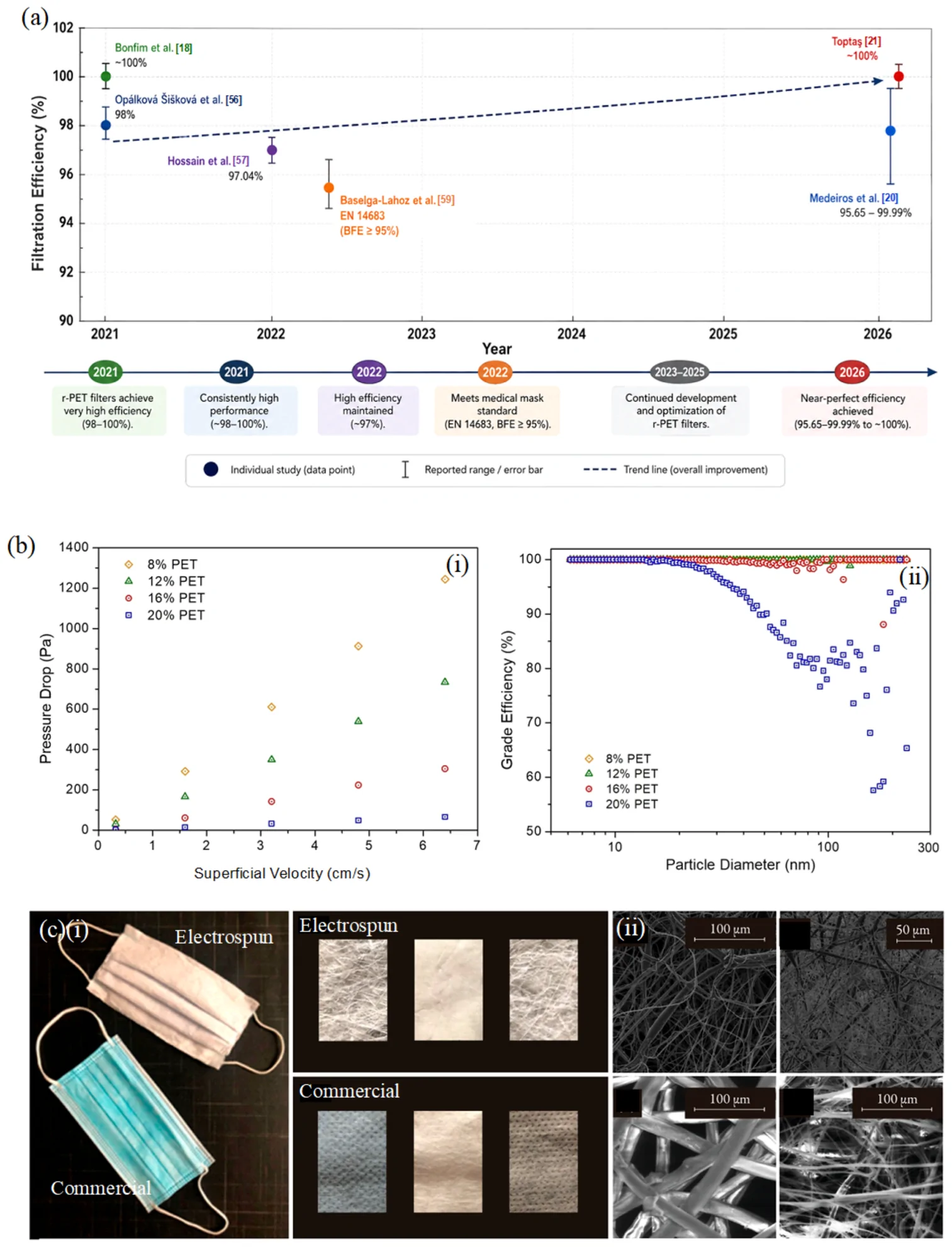
Recent developments in electrospun r-PET air filtration materials and their applications. (**a**) Timeline illustrating the evolution of filtration efficiency reported for electrospun r-PET membranes from 2021 to 2026 [18,20,21,56,57,59]. (**b**) Effect of PET concentration on (i) pressure drop and (ii) particle filtration efficiency of electrospun PET nanofiber filters [20]. (**c**) (i) Structural comparison between an electrospun PET-based surgical mask and a commercial surgical mask, including (ii) SEM micrographs comparing fiber morphology of the electrospun r-PET-based mask (top) and the commercial surgical mask (bottom): external layer (top-left, 100 µm) and filter layer (top-right, 50 µm) of the r-PET mask, and external layer (bottom-left, 100 µm) and filter layer (bottom-right, 100 µm) of the commercial mask [59].

The conversion of post-consumer PET waste into electrospun nanofibrous air filters represents one of the most strategically coherent expressions of circular economy thinking in filtration materials research. This approach simultaneously addresses the global plastic waste burden and the growing demand for sustainable, high-performance filtration media. Table 2 summarizes the recent progress in r-PET membranes for air filtration and their major findings. Bonfim et al. [18] demonstrated electrospun waste PET bottles dissolved in TFA/DCM into standalone filter membranes characterized by mechanical resistance of 4 MPa, porosity of 96%, and collection efficiency approaching 100% at a face velocity of 4.8 cm/s and pressure drop of 212 Pa. The same group subsequently reported that reducing fiber diameter from 3.25 µm to 0.65 µm simultaneously increased the tensile strength from 3.2 to 4.5 MPa while achieving collection efficiency of up to 99% at a substantially lower pressure drop of 19.4 Pa [44]. Those findings established that structural refinement in r-PET membranes simultaneously improves both mechanical integrity and filtration quality. Opálková Šišková et al. [56] applied r-PET from domestic plastic waste in HFIP/DCM to produce nanofibrous membranes with an average fiber diameter of 95 ± 37 nm, retaining particles of approximately 120 nm with more than 98% filtration efficiency while achieving 94% water vapor permeability, a combination of high particle capture and breathability particularly relevant for personal protective equipment. Hossain et al. [57] fabricated r-PET membranes from bottle chips with uniform fibers averaging 198.5 nm in diameter, achieving 97.04% filtration efficiency for 0.3 µm particles in compliance with ISO 16890 [58], with the membrane demonstrating water repellence, washability, and reusability. The continuous improvement in filtration performance achieved by electrospun r-PET membranes from 2021 to 2026 is summarized in Figure 2a.

A systematic parametric optimization was provided by Medeiros et al. [20]. They applied a fractional factorial design to quantify the effects of polymer concentration, flow rate, and applied voltage on r-PET membrane performance. The specific optimal condition at 16 wt% of r-PET, 1.2 mL/h, and 22 kV yielded uniform fibers with an average diameter of 328.6 nm and collection efficiencies of 95.65–99.99%, with theoretical porosity ranging from 96.47% to 98.73% across formulations. Figure 2b summarizes the effects of PET concentration and electrospinning conditions (flow rate, applied voltage, and tip-to-collector distance) on pressure drop and particle filtration efficiency, highlighting the importance of fiber diameter optimization. This work is significant because it moves beyond single-condition demonstrations toward process understanding capable of underpinning reproducible scale-up. At the device level, Baselga-Lahoz et al. [59] developed a scalable method for manufacturing surgical masks entirely from electrospun r-PET fibers, characterizing filtration performance against submicron aerosolized particles and microparticulate matter under conditions simulating actual mask use, and validating performance against EN 14683 [60] medical mask standards. The multilayer structure and fiber morphology of the electrospun PET-based mask compared with a commercial surgical mask are shown in Figure 2c. This study demonstrates a product-level validation step that moves r-PET filtration beyond membrane characterization toward real-world application readiness.

Collectively, these studies demonstrate that electrospun r-PET nanofibrous membranes are not merely a sustainable substitute for virgin polymer filters, but a genuinely competitive filtration material capable of achieving high collection efficiency, adequate mechanical strength, and application-relevant breathability across both HVAC and personal protective equipment contexts. Despite these advantages, several challenges remain. For example, variations in waste feedstock composition, degradation during recycling, contamination, and solvent requirements may affect the reproducibility and quality of electrospun membranes [18]. This variability has a clear cause. Repeated melting and reprocessing break down PET’s polymer chains. Shorter polymer chains result in a lower-viscosity electrospinning solution, which affects fiber formation. Over four extrusion cycles, intrinsic viscosity drops from about 0.80 to 0.65 dL/g, and crystallinity rises from 23.0% to 29.5% [61]. For example, electrospun recycled PET has been reported to form fibers averaging 1.44 μm in diameter, compared with 0.52 μm for virgin PET spun under similar conditions. This difference in fiber diameter was attributed to the lower viscosity of the recycled polymer solution (180 cP) compared with that of the virgin PET solution (280 cP) [62]. Recycled feedstock can also carry over contaminants such as catalyst residues, colorants, adhesives, and label fragments, which further complicate solution preparation [63]. Inconsistent feedstock quality is a real, largely unaddressed source of variability in r-PET filter performance. Furthermore, comprehensive life-cycle assessments comparing virgin PET and r-PET filtration systems remain limited. Nevertheless, recycled PET represents one of the most promising material platforms for sustainable air filtration technologies [18]. Increasing attention should be directed toward engineering membrane morphology and architecture to maximize filtration performance while preserving the sustainability advantages offered by PET and r-PET feedstocks, which are discussed in the following section.

## 6. Morphology Engineering as the Key to High-Performance PET Based Filters

While the previous section established that PET is a credible and sustainable filtration feedstock, filtration performance is ultimately governed more by membrane morphology than by polymer chemistry. This distinction carries a direct practical implication: two membranes fabricated from identical PET feedstocks under different electrospinning conditions can deliver entirely different filtration outcomes. Electrospun nanofiber filters possess great potential for air filtration, provided the structural characteristics of the nanofibers are optimized to achieve the right balance between collection efficiency and pressure drop [47]. The growing recognition of this morphology-performance dependency has shifted the research focus from material selection towards deliberate structural engineering, where parameters such as fiber diameter, porosity, pore size distribution, surface morphology, and membrane architecture are tailored to maximize the QF [64]. For r-PET specifically, this paradigm shift is particularly consequential because the same waste feedstock can yield filtration media of substantially different quality depending entirely on how its morphology is engineered. The following subsections examine each structural variable critically and systematically.

### 6.1. Fiber Diameter: The Primary Morphological Handle

Fiber diameter is widely known as the most influential morphological parameter governing the filtration performance of electrospun membranes [65]. Unlike many structural variables that affect either filtration efficiency or pressure drop independently, fiber diameter directly influences both parameters simultaneously. Reducing fiber diameter increases the specific surface area available for particle capture, enhances interception and diffusion mechanisms, and promotes operation within the slip-flow regime [66]. Consequently, finer fibers generally exhibit superior particle removal performance compared with larger fibers. Numerous studies have confirmed the strong relationship between fiber diameter and filtration efficiency. For example, Bian et al. [67] reported that PM_2.5_ removal efficiency decreased from 98.61% to 60.06% as fiber diameter increased from 123.2 to 245.5 nm, while increasing filter thickness from 0.33 to 1.37 μm raised removal efficiency from 80.72% to 97.79%; packing density, by contrast, showed no clear correlation with performance (correlation coefficient 0.23–0.27). Their findings demonstrated that fiber diameter and filter thickness must be optimized together to achieve balanced filtration performance. Similarly, Kamil et al. [68] reported that fiber diameter follows a power-law relationship with the electrospinning flow-rate-to-current ratio, with exponents of 0.535 for polyacrylonitrile, 0.440 for poly(lactic acid), and only 0.066 for acrylonitrile butadiene styrene. These findings indicate that the influence of this ratio on fiber diameter is polymer-specific rather than universally applicable across different materials. Their optimized double-layer PLA-ABS membrane, combining fiber diameters of approximately 771 nm and 1086 nm, respectively, achieved a filtration efficiency above 95.56% at a pressure drop of 61.73 Pa and a quality factor above 0.052 Pa^−1^ at a face velocity of 5.3 cm s^−1^.

The importance of fiber diameter is particularly evident in electrospun r-PET membranes. Medeiros et al. [20] demonstrated that controlling electrospinning parameters such as polymer concentration, applied voltage, and flow rate enabled the production of uniform nanofibers with enhanced filtration performance. Likewise, Bonfim et al. [18] reported that reducing fiber diameter from 3.25 to 0.65 μm significantly improved particle collection efficiency, reaching approximately 99% while maintaining a relatively low pressure drop of only 19.4 Pa. Their findings clearly demonstrate that fiber diameter optimization is a critical design strategy for improving the performance of r-PET filtration membranes. However, the relationship between fiber diameter and filtration performance is not linear. While smaller fibers generally improve particle capture, excessive diameter reduction can produce densely packed membrane structures that significantly increase airflow resistance. Under such conditions, the gains in filtration efficiency may be offset by disproportionate increases in pressure drop. Liu et al. [69] highlighted this limitation in their review on electrospun air filtration materials, concluding that decreasing fiber diameter alone does not necessarily improve overall filter performance. Instead, fiber diameter must be balanced against membrane porosity, packing density, and thickness to achieve an optimal quality factor.

Therefore, the objective of morphology engineering should not aim to minimize fiber diameter indiscriminately. Rather, an optimal diameter range should be identified that maximizes particle capture while maintaining acceptable airflow resistance. This distinction is particularly important for r-PET membranes, where the filtration performance depends on balancing enhanced interception and diffusion mechanisms with the pressure-drop penalties associated with dense fiber packing [7]. Consequently, fiber diameter optimization should be considered as part of an integrated membrane design strategy rather than an isolated parameter adjustment.

### 6.2. Porosity and Pore Size Distribution

While fiber diameter governs particle–fiber interactions at the microscopic level, membrane porosity and pore structure determine how air travels through the filtration medium. Together, these parameters influence airflow resistance, particle transport, and the probability of particle capture [66]. Compared with conventional microfiber filters, electrospun nanofibrous membranes typically exhibit high porosity, interconnected pore networks, and small pore sizes [46]. These characteristics allow efficient particle removal while maintaining relatively low pressure drops, making electrospun membranes attractive for air filtration applications [46]. Porosity and pore size are closely related to fiber diameter and fiber packing density. In randomly oriented electrospun membranes, average pore size generally decreases as fiber diameter decreases. Previous studies have reported that maximum pore size may reach approximately ten times the fiber diameter, indicating that fiber diameter control can be used as an effective strategy for tailoring membrane pore structures [70]. However, average pore size alone does not fully describe membrane performance.

Pore size distribution may be equally important. Membranes containing a broad or hierarchical pore structure can provide multiple airflow pathways, allowing air to pass through larger pores while maintaining effective particle capture in smaller pore regions. Such structures may improve airflow permeability without significantly compromising the filtration efficiency [71]. Consequently, the design of pore architecture has emerged as an important aspect of morphology engineering. The importance of pore structure was highlighted by De Riccardis [72], who reported that nanofibrous membranes with more uniform fiber distributions achieved filtration efficiencies as high as 99.78% for particles in the 300–500 nm range. Interestingly, the best performance was not necessarily obtained from membranes containing the smallest fibers. This means that uniform pore distribution and controlled membrane architecture can independently influence filtration performance, apart from the effect of fiber diameter. The growing importance of pore architecture is further illustrated by recent multilayer filtration studies. Razbin et al. [73] demonstrated that optimizing pore size distribution across a bilayer membrane using artificial neural networks and genetic algorithms resulted in a filtration efficiency of 99.94% with a pressure drop of only 40 Pa. Compared with conventional single-layer designs, bilayer structures provide greater flexibility for balancing filtration efficiency and airflow resistance. These findings suggest that future advances in r-PET filtration membranes may depend less on reducing pore size and more on engineering hierarchical pore structures capable of simultaneously enhancing airflow transport and particle capture. The ability to control pore architecture through morphology engineering provides new opportunities for improving filtration performance and forms the foundation for advanced structures such as bead-on-string and bilayer filtration membranes discussed in the next sections.

### 6.3. Bead-on-String Architecture: From Defect to Deliberate Design

An important development in the design of electrospun membranes has been the transformation of bead-on-string structures from perceived defects into effective filtration architectures. Traditionally, bead formation was regarded as undesirable because it indicated insufficient polymer chain entanglement or unstable electrospinning conditions. The cause of bead formation is attributed to Rayleigh instability, whereby the surface tension of the polymer jet is greater than the viscoelastic force of the polymer, according to Xue et al. [74]. If the concentration of polymer is low, weak chain entanglement allows the jet to break into droplets or bead-like structures. As polymer concentration increases, the bead morphology progressively changes from spherical to spindle-shaped and finally to uniform fibers. For many years, research on electrospinning was primarily directed toward the elimination of beads to produce smooth and uniform nanofibers [75].

**Figure 3:**
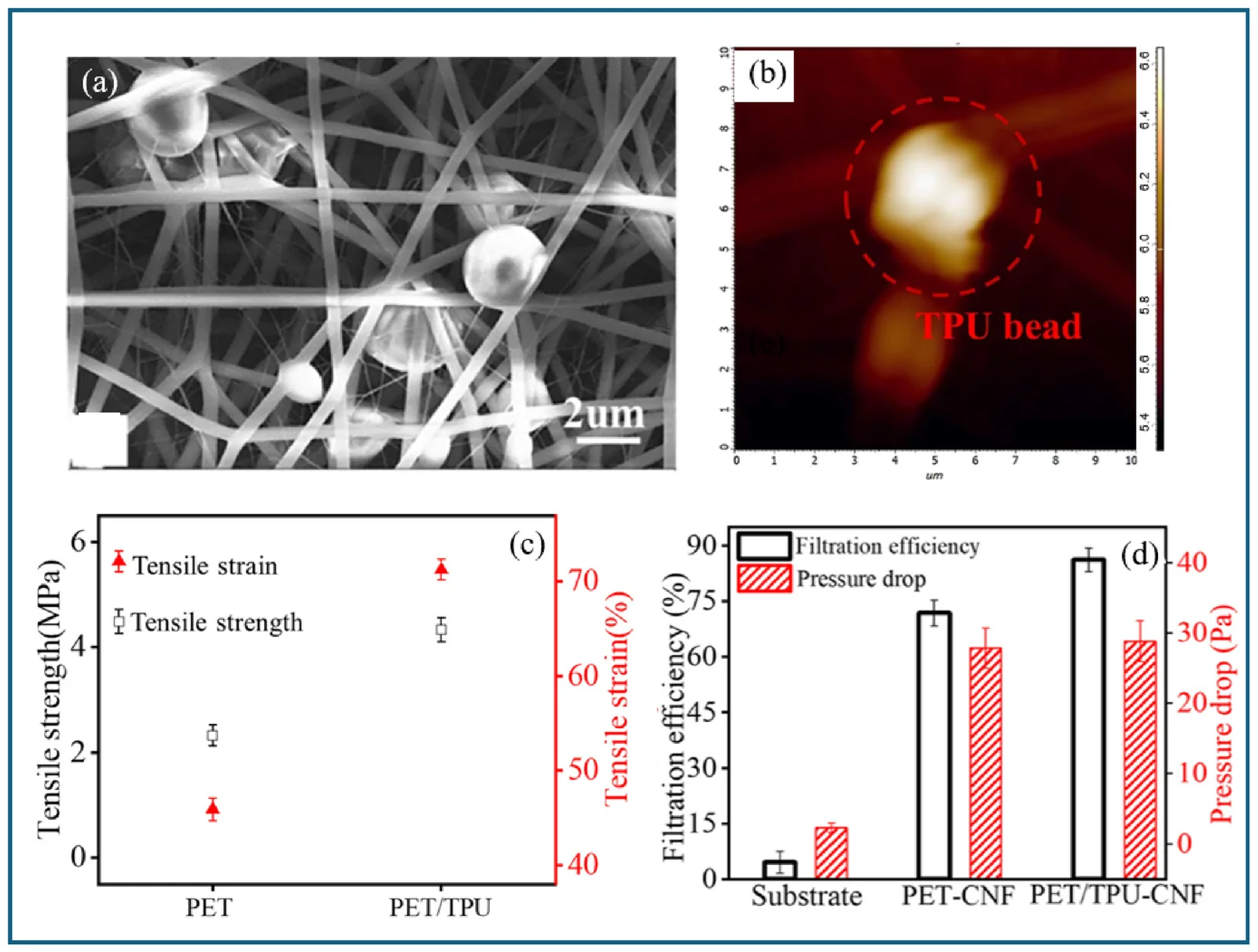
(**a**) SEM image of the bead-on-string morphology of the PET/TPU membrane, (**b**) AFM image showing the formation of TPU beads interconnected with PET fibers, (**c**) tensile strength and tensile strain, and (**d**) filtration efficiency and pressure drop of CNF, PET-CNF, and PET/TPU-CNF membranes [80].

However, recent studies have demonstrated that bead-on-string structures possess special structural properties that are beneficial for air filtration applications. These structures are not defects, but rather represent a hierarchical structure of large bead regions and very small fiber segments. This is an additional level of design flexibility over what can be achieved using uniform fibers [76,77]. The filtration advantages of bead-on-string membranes are a result of the synergistic filtering effect of the bead and string. Bead-on-string structures not only exhibited high filtration efficiency but also had lower pressure drop than conventional nanofibrous membranes. The larger area of the bead increases local porosity which increases the air flow throughput and decreases the resistance to air flow through the bead. In contrast, the ultrafine fiber segments located between adjacent beads provide a high specific surface area for particle capture through interception and diffusion mechanisms. In addition, the bead structures themselves may contribute to inertial impaction because of the interaction between the particle and the bead. Consequently, bead-on-string membranes can achieve a better balancing of filtration efficiency and pressure drop compared with many other conventional nanofiber architectures [78]. Computational modeling has also been employed to assist in the understanding of the performance advantages of bead-on-string structures. A recent simulation study [79] found that bead shape factor (length-to-height ratio) typically ranges from 1 to 4 and bead size factor (bead height to fiber diameter ratio) typically ranges from 2 to 15. Within this range, an elliptical bead with a shape factor of 1.5 and a size factor of 2.0 gave the highest QF. Applying this geometry to a virtual fiber model identified an optimal bead volume fraction of approximately 30%, which reduced pressure drop by 32% relative to a bead-free membrane while lowering particle capture efficiency by only 11% at the MPPS. This was attributed to the three-dimensional airflow field generated around the bead–string geometry, which reduced pressure drop while preserving particle capture efficiency. This study establishes how bead volume fraction and relative bead size influence performance; it describes average behavior across different samples rather than variability within one [79]. In practice, individual membranes are not perfectly monodisperse. PET/TPU bead-on-string membranes have been reported with an average bead diameter of 2050 ± 900 nm, a spread of around 44% [80]. Since Zhang et al.’s modeling showed that the quality factor peaks at a moderate bead-to-fiber size ratio, while filtration efficiency decreases sharply at high bead loading (80.5% to 54.2%) [79], a single membrane containing substantial size variation would be expected to exhibit a combination of these regimes across different areas of the membrane. This could create locally lower-resistance regions that concentrate particulate loading and promote non-uniform clogging, shortening filter life relative to a narrower distribution [24,81,82]. Bead-on-string PET/PVA nanofibrous membranes have demonstrated filtration efficiencies of 99.87–99.89% for NaCl and DEHS aerosols at pressure drops of 160.1–165.3 Pa alongside a tensile strength of 4.91 MPa. These beads on the membrane play a critical role in determining the overall performance. The hierarchical architecture provides enlarged pore channels that promote airflow while maintaining sufficient fiber surface area for particle capture, thereby enabling excellent filtration efficiency with moderate pressure drop and good mechanical strength [75]. The second example was reported by Guo et al. [80] who fabricated PET/TPU composite nanofiber filters through a one-step co-electrospinning process. The membrane obtained showed a filtration efficiency of 83.64% under a pressure drop of just 28.96 Pa at a face velocity of 5.3 cm s^−1^. The composite membrane exhibited better filtration performance and had a tensile strength of 4.33 MPa and an interfacial adhesion strength of 1.385 N cm^−1^. Notably, the bead-on-string morphology generated during co-electrospinning contributed to both filtration efficiency and mechanical robustness. As illustrated in Figure 3a,b, the incorporation of TPU beads interconnected by fibers generated characteristic bead-on-string structures that acted as bonding nodes between PET fibers and cellulose nanofibers (CNFs), thereby enhancing interfacial interactions and mechanical integrity. The PET/TPU-CNF membrane had significantly higher tensile strength and elongation at break compared with pristine PET membranes (Figure 3c), while maintaining superior filtration performance with only a moderate increase in pressure drop (Figure 3d).

Therefore, bead-on-string structures should no longer be considered as electrospinning defects. Rather, they are a conscious morphology-engineering approach that can enhance the balance between filtration efficiency and pressure drop. The ability to independently manipulate bead geometry, fiber diameter, and pore structure provides new opportunities for designing high-performance r-PET filtration membranes. Moreover, the hierarchical nature of bead-on-string architectures provides a natural foundation to extend the architecture to more complex multilayer and bilayer filtration systems, which are discussed in the following section.

### 6.4. Spider-Net Structures

Spider-net structures are another morphology of interest for air filtration. Their interconnected ultrafine fibers can increase specific surface area and provide numerous interception and diffusion sites while maintaining interconnected airflow channels. From a morphology-engineering perspective, spider-net structures are particularly relevant because their filtration behavior is governed by network density, fiber junction and pore hierarchy rather than fiber diameter alone.

Self-assembled 2D polyvinyl butyral (PVB) nano-networks were successfully engineered on PVDF substrates through a hierarchical phase-separation strategy. This membrane has good surface polar functional groups in PVB nanowires with an average diameter of approximately 31 nm, to enhance particulate matter capture through dipole–dipole interactions and hydrogen bonding. These PVB-based membranes achieve a PM_0.3_ capture efficiency of 99.6% while maintaining a low pressure drop of 58.8 Pa and a quality factor of 0.095 Pa^−1^ [83].

Another approach uses the electronetting technique to produce continuous PAN nanonets with diameters around 30 nm. These structures have very small pore sizes of 0.26 µm and high porosity of 92.1%, achieving 99.996% removal efficiency for PM_0.3_ with a pressure drop of 73.5 Pa and a quality factor of 0.14 Pa^−1^. The performance of these nanonets is further enhanced by the “slip effect” of air molecules on the surface of ultrafine fibers, which significantly reduces aerodynamic drag compared to conventional fibers [84].

Similarly, biodegradable PLA membranes with spider-web, multi-scale nanofibers and bulky beads were produced in a one-step process using a double-jet synchronous electrospinning technique. These membranes achieve PM_0.3_ filtration efficiency of 99.87%, a low pressure drop of 19 Pa and a high quality factor of 0.321 Pa^−1^. The bulky bead in this structure improves structural fluffiness and increases the membrane porosity to 94.65%, thereby facilitating efficient particle capture with minimal airflow resistance [85].

**Figure 4:**
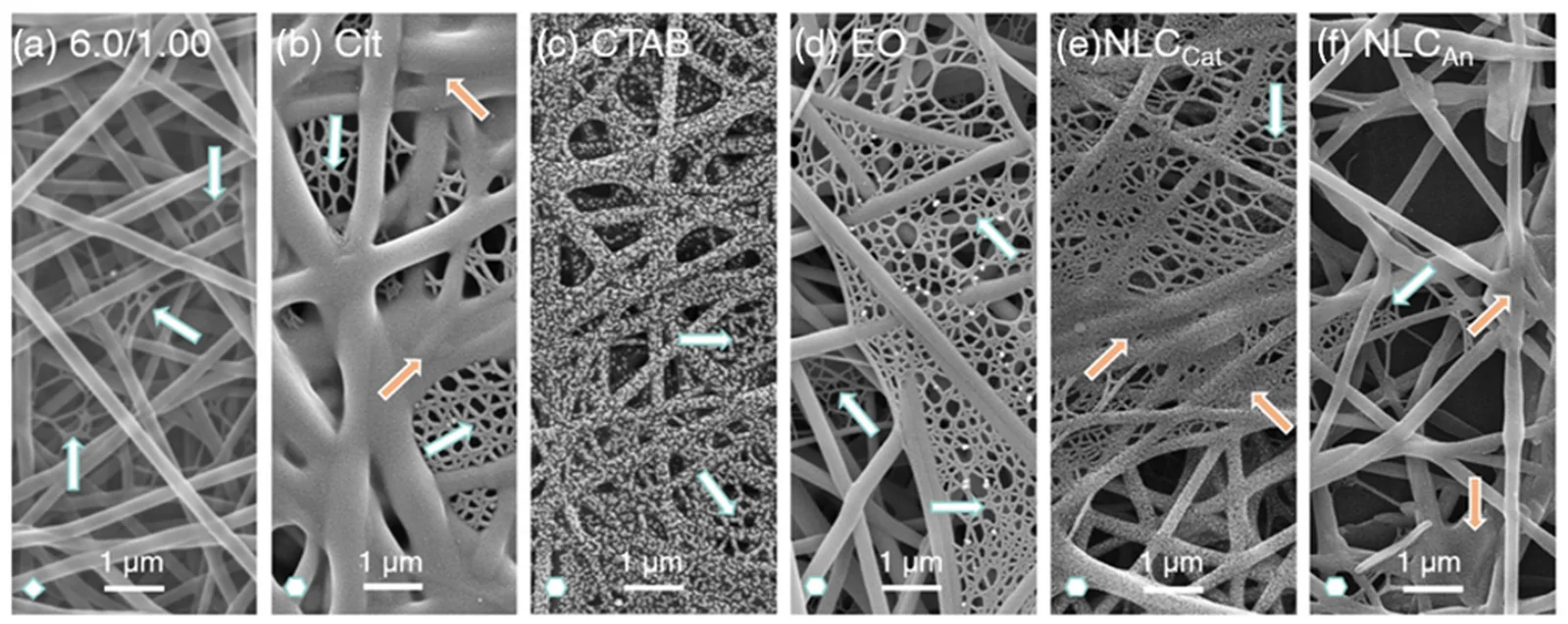
Representative SEM images showing the evolution of spider-net morphology in PVA/Chitosan nanofibrous membranes with different additives: (**a**) 6.0% PVA and 1.00% CS without additive, (**b**) citric acid, (**c**) cetyltrimethylammonium bromide (CTAB), (**d**) pure *Lippia sidoides* essential oil, and the essential oil encapsulated as (**e**) a cationic nanostructured lipid carrier and (**f**) an anionic nanostructured lipid carrier [86].

Furthermore, spider-net structures based on PVA/chitosan (CS) blends have been optimized through the incorporation of additives such as citric acid and essential oils. As shown in Figure 4, the PVA/chitosan membrane exhibits interconnected nanofibrous networks, while the addition of citric acid, CTAB, and *Lippia sidoides* essential oil produces distinct changes in network density, fiber arrangement, and the formation of secondary nanoscale structures. The use of essential-oil-based nanostructured lipid carriers further modifies the spatial distribution and coverage of the spider-net network, demonstrating that changes in solution composition and phase behavior can be exploited to tailor the hierarchical morphology of electrospun membranes. Such morphological control can increase the available surface area and particle-capture sites while preserving interconnected pathways for air transport. Notably, the optimized essential-oil-containing membranes achieved a filtration efficiency of 99.6% and a quality factor of 0.048 Pa^−1^, highlighting the potential of additive-assisted spider-net architectures for achieving efficient particulate capture with relatively low airflow resistance [86].

Collectively, the aforementioned studies demonstrate that spider-net structures can improve particle capture while maintaining a low pressure drop through the synergistic effects of ultrafine fibers, high porosity, and tailored network morphology.

### 6.5. Bilayer and Multilayer Architectures: Extending Morphology Engineering Beyond the Single Fiber

The bead-on-string structures improve filtration performance at the fiber level, while bilayer and multilayer architectures address the efficiency at the membrane level. The fundamental concept is to divide different filtration functions into multiple layers having complementary structural characteristics. Typically, a coarser upstream layer captures larger particles through inertial impaction and interception, whereas a finer downstream layer removes submicron particles through diffusion and electrostatic interactions [87]. This graded filtration strategy reduces premature clogging of the fine-fiber layer, improves dust-holding capacity, and distributes pressure drop throughout the membrane thickness rather than concentrating it within a single dense layer [88]. The effectiveness of multilayer filtration systems has been demonstrated across a wide range of fibrous filter materials. Multilayer membranes offer a higher degree of flexibility in filtration efficiency–pressure drop, mechanical stability, and service lifetime than single-layer membranes. More importantly, the independent design of each layer makes it possible to create membrane structures to meet particular filtration requirements [89].

Liu et al. [90] fabricated hierarchical micro/nanofibrous filters by combining electrospun polyurethane (PU) nanofibers with PET microfibers through spunlace reinforcement. The resulting composite membrane achieved a PM_0.26_ filtration efficiency of 76.63% at a pressure drop of only 17.3 Pa, corresponding to a quality factor of 0.084 Pa^−1^. The optimum performance was obtained at a PU nanofiber loading of 1.87 wt% relative to the PET microfiber scaffold. Lower loadings of 0.62 and 1.25 wt% resulted in PM_0.26_ efficiencies of only 35.53% and 59.67%, respectively, whereas increasing the loading to 2.5 wt% further improved the efficiency to 86.25% but increased the pressure drop disproportionately to 26.4 Pa. These results indicate that a loading of 1.87 wt% provides the best balance between filtration efficiency and pressure drop. The PET microfiber framework did not merely provide mechanical support. Instead, it was actively involved in capturing particles and blocking larger particles from contacting the PU nanofiber layer. To gain further understanding of the filtration mechanism, the authors performed three-dimensional (3D) numerical simulations of particle transport and deposition within the hierarchical filter structure. The simulated filtration processes and particle distributions proved that the hierarchical PET/PU structure transformed the filtration mechanism from conventional deep-bed filtration observed in PET microfibers to a surface-dominated filtration mode. The PET microfibers served as a highly porous support layer that facilitated airflow and captured coarse particles, whereas the PU nanofibers formed a fine filtration layer that efficiently retained smaller particles through interception and Brownian diffusion. Consequently, particle accumulation was concentrated near the upstream surface rather than throughout the filter thickness, resulting in lower pressure drop, improved filtration efficiency, and enhanced filter regeneration ability. These simulation results demonstrate that multilayer micro/nanofibrous architectures successfully harness the complementary functions of each layer, for improved particle capturing and airflow resistance.

**Figure 5:**
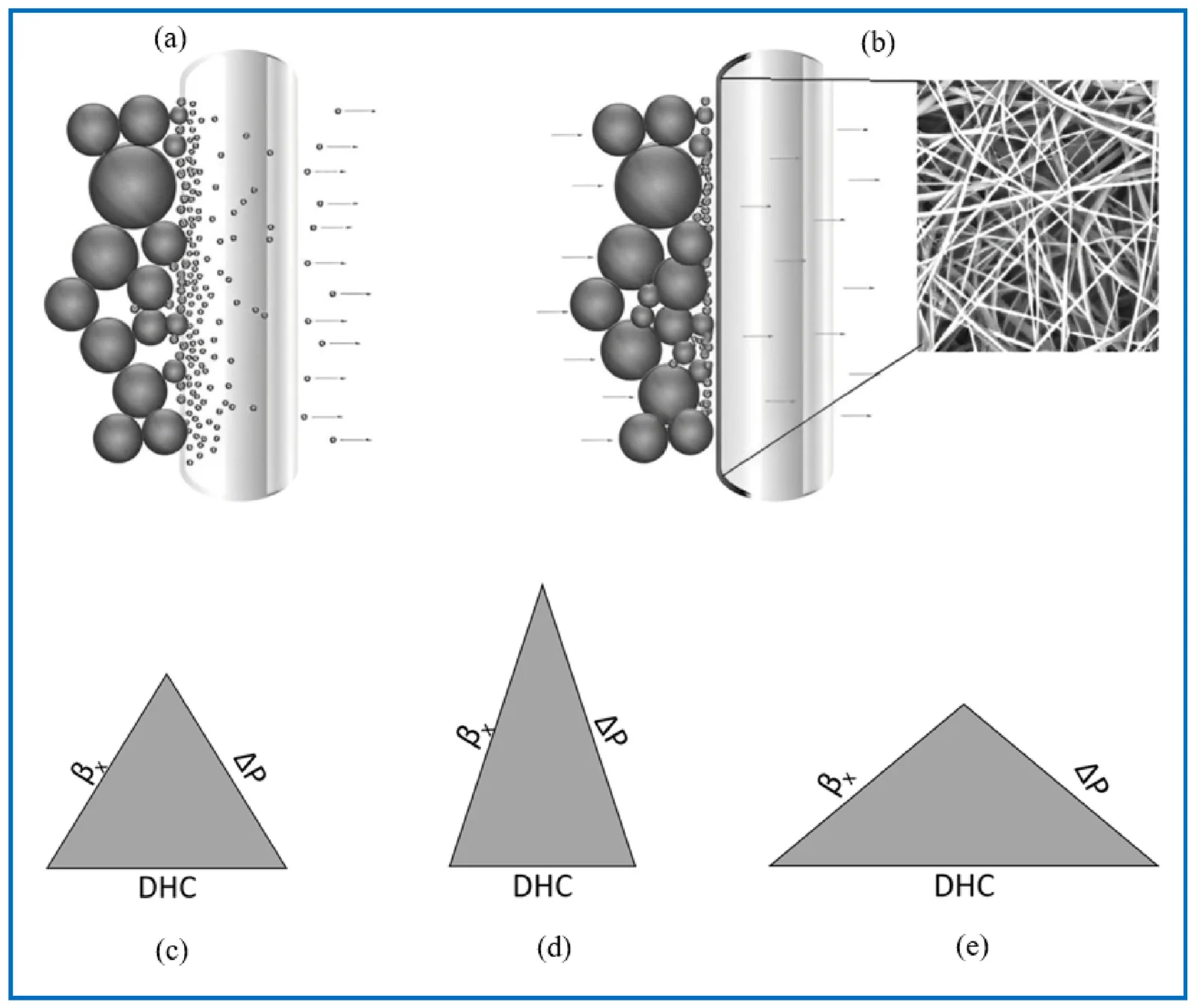
Influence of layer arrangement on filtration mechanisms and performance trade-offs in multilayer filters. Schematic illustrations show (**a**) depth filtration and (**b**) surface filtration resulting from different placements of the electrospun nanofiber layer, with arrows indicating the direction of fluid flow. (**c**–**e**) Corresponding β_x_-ΔP-DHC triangles illustrate the trade-off among filtration efficiency (β_x_), pressure drop (ΔP), and dust-holding capacity (DHC) when prioritizing different performance parameters [91].

Recent studies have further shown that the success of multilayer filtration systems is not only related to the number of layers, but also to the structural characteristics of each layer. Membrane performance is determined by a combination of parameters such as fiber diameter, basis weight, porosity, pore size distribution, and layer sequence. Weiter et al. [91] demonstrated that the filtration efficiency–pressure drop, and dust holding capacity of the nanofiber layer were greatly affected by the nanofiber modification of glass-fiber substrates. As illustrated in Figure 5a,b, the arrangement of the nanofiber layer determines whether the filter predominantly operates through depth filtration or surface filtration. When the nanofiber layer is placed on the upstream side, particles are mainly retained on the surface; therefore, facilitating backwashing will be easier and filter lifetime can be extended. In contrast, positioning the nanofiber layer downstream enables the coarse substrate to utilize its full volume for particle capture, thereby preserving the depth filtration characteristics but increasing flow resistance. Furthermore, the β_x_–ΔP–DHC relationship shown in Figure 5c–e highlights the inherent trade-off among filtration efficiency (β_x_), pressure drop (ΔP), and dust-holding capacity (DHC). Therefore, besides material selection, rational layer arrangement provides an additional degree of freedom for balancing these competing parameters. These findings have important implications for the development of r-PET filtration membranes. They propose that filtration performance can be enhanced not only by optimizing fiber morphology through electrospinning but also by rationally designing the sequence and structural function of each membrane layer. Current research has largely focused on optimizing electrospinning parameters or fiber morphology within single-layer membranes. Multilayer architectures however offer an extra degree of structural control which can potentially further enhance filtration performance. For instance, a bead-on-string r-PET layer could act as a highly porous upstream pre-filtration layer, whereas a finer straight-fiber r-PET layer could be used as the main particle-capturing layer. This setup can enhance dust-holding capacity and reduce pressure-drop accumulation during operation. Thus, combining the bead-on-string structures, graded pore size distributions, and layer-specific functionalities has many potentials to address the conventional efficiency–pressure drop dilemma. Moreover, the new developments in the design of multilayer membranes could facilitate the transition of r-PET filtration materials from laboratory-scale demonstrations to practical HVAC and industrial air filtration applications.

### 6.6. Durability and Environmental Stability of r-PET Membranes

The long-term service life of r-PET filtration membranes depends on how well the membrane retains its mechanical and structural integrity during extended operation. The tensile strength of electrospun PET membranes increases from 3.2 to 4.0 MPa as fiber diameter decreases from 3.25 to 1.27 µm [18]. Denser fiber packing similarly improves the mechanical robustness of r-PET nonwovens [44]. This inverse relationship between fiber diameter and mechanical strength suggests that morphology-engineering strategies capable of producing finer, more uniformly packed fibers could improve membrane durability. For instance, the optimized bead-on-string and multilayer architectures discussed earlier may simultaneously enhance filtration performance and extend membrane service life.

Humidity is another factor relevant to service life. PET is a polyester containing ester linkages that are susceptible to hydrolytic chain scission in the presence of water. PET hydrolysis becomes appreciable primarily under conditions of elevated temperature and high relative humidity. An estimated activation energy of approximately 100 kJ mol^−1^ across a wide range of conditions (40–100% RH, 60–160 °C) [92] is reported for PET. As chain scission progressively reduces the molecular weight of PET, hydrolytic aging can lead to a measurable decline in its mechanical properties over time [93]. For r-PET filters operated under typical indoor HVAC conditions, where temperatures generally remain well below PET’s glass transition temperature, hydrolytic degradation over the membrane’s service life is expected to be limited. However, the stability of r-PET membranes under sustained high humidity and elevated temperature, as may occur in industrial environments or tropical climates, should be evaluated further. Humidity may also affect electrostatically assisted capture more broadly, as accumulated charge in electret-type electrospun nanofibers has been shown to dissipate gradually under sustained high-humidity exposure [84], reducing particle-capture efficiency over time in r-PET membranes.

Thermal stability is comparatively well characterized for r-PET nanofibers. Thermogravimetric analysis of electrospun waste-PET-based nanofibers has demonstrated stability up to 150–160 °C [94], comfortably exceeding the operating range of standard HVAC and indoor air filtration (typically below 60 °C). r-PET therefore appears well suited for standard air filtration applications, whereas high-temperature exhaust and industrial filtration applications may require either an alternative polymer or a thermally stabilized r-PET composite. These durability considerations reinforce that r-PET’s mechanical, hydrolytic, and thermal behavior are each connected to the same morphological parameters discussed throughout this review—fiber diameter, packing density, and porosity—rather than being independent material constants. However, r-PET-specific accelerated-aging data under combined humidity and thermal cycling remain scarce. Future studies quantifying service-life degradation directly for bead-on-string and multilayer r-PET architectures, rather than extrapolating from bulk PET or other electret polymers, would strengthen predictive design of long-lasting r-PET filtration membranes.

## 7. Conclusions

In summary, recycled polyethylene terephthalate (r-PET) has emerged as a highly promising material for sustainable air filtration applications due to its abundance, low cost, mechanical robustness, and compatibility with circular economy principles. With the recent developments in electrospinning technology, waste PET bottles and textiles can be transformed into high-performance nanofibrous membranes with high filtration efficiency equivalent to that of conventional virgin polymer filters. This review highlights that r-PET filtration membranes, apart from the material properties, critically rely on the deliberate engineering of membrane morphology for optimization in air filter applications. The influence of structural parameters, including fiber diameter, porosity, pore size distribution, surface morphology, bead-on-string architecture, and multilayer configuration, govern the balance between filtration efficiency and pressure drop. Among these, bead-on-string structures and bilayer architectures are especially attractive solutions for overcoming the traditional efficiency–pressure drop trade-off that limits conventional filtration media. Further evidence reviewed indicates that morphology engineering could have more impact on filtration performance than polymer chemistry alone. Thus, future studies are required to focus on the rational design of membrane architectures that can achieve maximum QF while maintaining the sustainability benefits attributed to recycled feedstocks. In parallel, variability of feedstock, sustainability of solvent, large-scale production, and standardization of performance are among the challenges that should be solved to enable industrial implementation. To conclude, the valorization of waste, electrospinning technology, and advanced morphology engineering have great potential for sustainable high-performance filters. With continued development in scalable manufacturing and intelligent membrane design, r-PET nanofibrous membranes are expected to play an increasingly important role in next-generation HVAC systems, personal protective equipment, and industrial air filtration technologies.

## 8. Recommendations and Future Prospects

The electrospinning of r-PET filtration membranes has developed rapidly in recent years, and it has demonstrated that recycled polymers can be used as alternatives to conventional virgin materials. However, long-term progress will not come solely from the use of better materials. Rather, there is growing evidence that morphology engineering will have a greater impact on filtration performance. Therefore, the rational design of membrane architectures that can achieve both high filtration efficiency and low pressure drop is recommended for future research. In this review, some of the different methods of morphology engineering were listed, and one of the most promising was the bead-on-string structure. While recent studies have shown that they can enhance QF by reducing airflow resistance without compromising the efficiency of the particle-capturing ability, the relationship between bead geometry and filtration performance remains poorly understood. Future research should provide quantitative design criteria linking bead size, bead density, bead volume fraction, and fiber diameter to filtration efficiency–pressure drop, and dust-holding capacity. Such understanding would support the transition from empirical fabrication to predictive membrane design.

There is also substantial potential for further development of bilayer and multilayer filtration architectures. Current studies indicate that layered membrane structures can outperform conventional single-layer filters by distributing particle capture functions over multiple layers. However, the optimal combination of fiber diameter, fiber thickness, porosity, and layer sequence remains largely undiscovered for r-PET systems. The hierarchical combination of bead-on-string layers and fine-fiber capture layers should be prioritized in investigation to achieve higher QF and longer service lifetimes. The application of artificial intelligence (AI) is anticipated to become an increasingly important tool for filtration membrane development. AI-assisted optimization using genetic algorithms (GA) can support the development of r-PET air filters by directly connecting fabrication parameters to filtration outcomes, thereby enabling an optimal balance between particle capture and airflow resistance. Key morphological characteristics including fiber diameter, bead formation, pore size, and layer thickness are strongly influenced by fabrication parameters such as polymer concentration, voltage, flow rate, and deposition time. By integrating these parameters into a GA-based optimization framework, the electrospinning conditions can be systematically optimized to achieve the desired membrane morphology and filtration performance.

These structural features, in turn, influence the PM_2.5_ removal efficiency–pressure drop, and quality factor. By training models with experimental filtration data, AI models, such as artificial neural networks (ANN) and convolutional neural networks (CNN), can serve as high-fidelity surrogate models to predict the relationships between processing conditions, membrane morphology, and filtration performance. This enables the identification of membrane designs that have the best balance between high particle capture and low airflow resistance prior to fabrication, potentially reducing the number of experimental trials required. Furthermore, AI-based morphology analysis and prediction can be coupled with global sensitivity analysis to quantify the relative contributions of individual morphological characteristics to overall filter performance. This approach is particularly promising for bead-on-string and multilayer r-PET filters, where multiple processing and structural parameters must be optimized simultaneously.

Despite the promising performance of r-PET filters, sustainability challenges remain. Many of the current electrospinning processes are based on fluorinated solvent systems or solvents with high volatility. Thus, there is a need to study the development of greener solvent systems, solvent recovery technologies, and scalable manufacturing processes. In addition, standardized testing procedures should be developed to facilitate comparison between filtration studies and be applicable to industrial implementation. Overall, the future of r-PET air filtration depends on the combination of sustainable materials, innovative morphology engineering, and intelligent design strategies. The combination of bead-on-string architectures, multilayer membrane configurations, and AI-assisted optimization offers a promising pathway to break the traditional efficiency–pressure drop trade-off. With the improvement of these technologies, r-PET nanofibrous membranes are expected to play an increasingly important role in HVAC systems, personal protective equipment, and industrial air filtration applications. Ultimately, the integration of circular economy principles with advanced nanofiber engineering will be essential to transform recycled PET-based filtration technologies from laboratory-scale innovations into practical, sustainable solutions for next-generation air quality management.

## Figures and Tables

**Table 2:** Recent progress in r-PET membranes for air filtration and their major findings.

Reference	Feedstock	Fiber Morphology	Filtration Performance	Key Finding
Bonfim et al. [18]	PET bottle waste	Fiber refinement from micrometer to sub micrometre scale	Efficiency approaching 100%; pressure drop reported 19.4 Pa	Fiber refinement
Opálková Šišková et al. [56]	PET/silk-fibroin composite	95 ± 37 nm fibers	>98% efficiency	High capture and breathability
Hossain et al. [57]	Post-consumer PET bottle	198.5 nm fibers	97.04% efficiency	Water repellence and washability
Medeiros et al. [20]	r-PET	328.6 nm average fiber diameter	95.65–99.99% efficiency	Process optimization
Baselga-Lahoz et al. [59]	r-PET mask media	Multilayer application	>98.2% efficiency	Scalable mask fabrication
Toptaş et al. [21]	Recycled textile polyester	≈41 nm ultrafine fibers	Near-100% efficiency after electret charging	Electret enhancement

## Data Availability

No new data were created or analyzed in this study. Data sharing is not applicable to this article.
